# Measuring Alliance Toward Embodied Virtual Therapists in the Era of Automated Treatments With the Virtual Therapist Alliance Scale (VTAS): Development and Psychometric Evaluation

**DOI:** 10.2196/16660

**Published:** 2020-03-24

**Authors:** Alexander Miloff, Per Carlbring, William Hamilton, Gerhard Andersson, Lena Reuterskiöld, Philip Lindner

**Affiliations:** 1 Department of Psychology Stockholm University Stockholm Sweden; 2 Mimerse AB Stockholm Sweden; 3 Department of Behavioral Sciences and Learning Linköping University Linköping Sweden; 4 Centre for Psychiatry Research Department of Clinical Neuroscience Karolinska Institute Stockholm Sweden

**Keywords:** alliance, virtual reality, exposure therapy, automated treatment, psychometric, embodiment, virtual therapist, virtual coach, avatar, usability, presence, empathy

## Abstract

**Background:**

Automated virtual reality exposure therapies (VRETs) are self-help treatments conducted by oneself and supported by a virtual therapist embodied visually and/or with audio feedback. This simulates many of the nonspecific relational elements and common factors present in face-to-face therapy and may be a means of improving adherence to and efficacy of self-guided treatments. However, little is known about alliance toward the virtual therapist, despite alliance being an important predictor of treatment outcome.

**Objective:**

In this study, we aimed to evaluate the first alliance instrument developed for use with embodied virtual therapists in an automated treatment format—the Virtual Therapist Alliance Scale (VTAS)—by (1) assessing its psychometric properties, (2) verifying the dimensionality of the scale, and (3) determining the predictive ability of the scale with treatment outcome.

**Methods:**

A psychometric evaluation and exploratory factor analysis of the VTAS was conducted using data from two samples of spider-fearful patients treated with VRET and the help of an embodied, voice-based virtual therapist (n=70). Multiple regression models and bivariate correlations were used to assess the VTAS relationship with treatment outcome, according to self-reported fear and convergence with presence and user-friendliness process measures.

**Results:**

The VTAS showed a sound two-factor solution composed of a primary factor covering task, goal, and copresence; adequate internal consistency; and good convergent validity, including moderate correlation (*r*=.310, *P*=.01) with outcomes over follow-up.

**Conclusions:**

These preliminary results suggest that alliance toward a virtual therapist is a significant predictor of treatment outcome, favors the importance of a task-goal over bond-factor, and should be explored in studies with larger sample sizes and in additional forms of embodiment.

## Introduction

The relationship between therapist and patient during psychotherapy, referred to as alliance and measured by a number of instruments such as the Working Alliance Inventory (WAI) [1], is considered an important common factor shared by diverse treatments [2]. Alliance is conceptualized as requiring three distinct processes: agreement on therapeutic goals, consensus on tasks that make up therapy, and bond between therapist and patient [3]. Alliance is also understood to occur in stages, first with identifying the therapist as a source of encouragement, warmth, and support and, in later stages, the development of a client’s faith and investment in the therapeutic process, a collaborative *working together* [4]. Some alliance measures, such as the Helping Alliance Questionnaire (HAq) [5], are an authoritative operationalization of their authors’ key theories; however, the boundaries of the popular WAI were never formalized [2] and researchers continue to refine the measure as they seek to develop a holistic understanding of alliance for their particular treatment.

Virtual reality exposure therapy (VRET) is a technology-based method of delivering exposure-based treatments; it has been tested in clinical trials for some 20 years, with positive results [6], but has only recently become available to consumers [7]. VRET works by using head-mounted displays to present computer-animated graphical stimuli or 360-degree video, interactive to head movements, while occluding the outside world [8]. Few studies, however, have assessed alliance during VRET treatments, perhaps due to an expectation that alliance will be poor in treatments that prevent face-to-face contact with a therapist [9]. Contrary to this expectation, studies of patients treated with VRET and in vivo exposure therapy for social anxiety disorder found no significant difference between interventions as measured by the WAI [10-12]. Using augmented reality exposure therapy (ARET), a related technology [13], the same lack of difference in WAI scores was found following treatment for small-animal phobia [14]. In a systematic review of VRET process and outcome research, only two studies were identified that captured alliance information [15]: a study of fear of flying, but not an acrophobia study, found a significant correlation with outcome.

Automated VRET treatments—replacing in vivo stimuli with virtual stimuli in addition to replacing human therapist with virtual therapist [16]—are an example of autonomous digital treatments, and being self-guided may be among the most scalable of psychological interventions [17,18]. However, previous research suggests that treatments conducted without therapist support suffer from lower adherence and efficacy as compared to treatments that include contact [19,20]. There is also evidence that more contact and better contact (ie, frequency of interactions and persuasive design elements, such as dialogue support) may result in more patients completing treatment [21]. Therefore, simulating key elements of face-to-face therapist interaction with a virtual agent manifesting visually and/or through auditory instruction may improve adherence and bridge the efficacy gap between guided and unguided interventions [22]. This is particularly so if they are successful in replicating common factors in therapy and nonspecific relational elements, such as empathy and therapist attention [23], which some have suggested are better conceived of as an active ingredient and should be intentionally used in treatment to facilitate better outcomes [24]. In low-income regions, they may also offer an opportunity to disseminate evidence-based cognitive behavioral therapy (CBT) where literacy skills are low and even therapist-supported online treatments are not appropriate [25].

To date, three automated VRET treatments have been tested in clinical trials: two for use with acrophobia [26,27] and one for use with spider phobia [28]. A treatment for social anxiety disorder has also been tested as a small pilot study [29]. Freeman et al [27] used what they referred to as a virtual coach, physically embodied in virtual reality (VR) by a trained actor, to assist patients to conduct behavioral experiments, challenge safety behaviors, repeat key learning points, and provide empathic encouragement. In the study by Donker et al [26], patients were given background information about phobias, provided case examples, and given motivation using a 2D, animated, mobile phone app-based avatar called *Tara*. The Hartanto et al [29] system enabled interactive dialogue with an animated virtual health agent via laptop computer to instruct in the use of VRET sessions; provide monitoring data interpretations, based on heart rate, for example; and motivate clients while completing treatments at home. However, the paradigm relied on a personalized treatment plan created by a human therapist versus the three more recent trials, which incorporated game-level-like progression systems similar to an exposure fear hierarchy. We designed our automated treatment for spider phobia [28] with a virtual therapist to deliver guidance and support from within the VRET application using primarily voice-based instructions and, in one version, a graphical representation. Participants were greeted from the beginning of treatment by the virtual therapist, provided information on the purpose of treatment, provided instructions on how to conduct exposure tasks, and were followed throughout treatment with encouragement and positive reinforcement when a task was completed.

To our knowledge, no clinical trial conducted using an automated VRET treatment with a virtual therapist has reported on alliance, despite the therapist-like human qualities their creators designed them with. There are indications, however, that alliance may be possible toward a virtual therapist. One recent trial of an automated online CBT program for insomnia [30] incorporated an audio-based avatar to guide patients through the application and to provide cognitive restructuring for sleep-related concerns. The authors identified comparable or higher goal and task subscale scores using an adapted WAI scale as compared to a therapist-led, Internet-based CBT (ICBT) program for tinnitus and similar task subscale scores as compared to an outpatient treatment group, but lower goal. Affective bond was stable and relatively high over six sessions according to five items from the Bern Post-Session Report. The authors suggested that a questionnaire designed specifically for automated programs and avatars is needed to provide a clearer picture of alliance relationship with symptom change. Correlations with outcome were found but only for the affective bond subscale.

Just one effort has been directed toward developing a WAI scale intended specifically for VRET and ARET treatment: the WAI applied to virtual and augmented reality (WAI-VAR) [31]. The WAI-VAR collected data from across three studies (n=75) for fear of flying, adjustment disorder (VRET), and cockroach phobia (ARET). Clinically significant *recovered* and *improved* participants had large effect size differences with *not changed* patients according to the WAI-VAR (eta-square=0.32, *P*<.001) and large Spearman correlations were noted between alliance and clinically significant change (*r*=.55, *P*<.001). High scores were also found on the non-VR-specific WAI-short form (WAI-S) [32].

The WAI-VAR [31], however, was developed to address comfort and trust with the virtual environment itself, replacing “my therapist” with “virtual environment” for all items of the Spanish version of the WAI-S. Recent research suggests that using conventional measures and simply replacing “therapist” with another term (eg, “app”) may be insufficient for accurately capturing the subtleties of therapeutic alliance [33]. Given the embodied nature of the virtual therapist in this automated treatment, we developed a novel instrument entitled the Virtual Therapist Alliance Scale (VTAS) to gather information on the patient relationship with the virtual therapist themselves. In addition to phrasing WAI items in terms of “the virtual therapist,” modified items from an empathy questionnaire [34] and copresence scale (ie, “the experience of being with another”) [35] were included to capture additional relational characteristics of working with the virtual therapist. A preliminary psychometric evaluation was conducted using data collected from two independent studies (Miloff et al [28] and Lindner et al, under review), convergent validity assessed according to common process measures (ie, presence and user friendliness), and the scale compared to treatment outcome (ie, self-reported fear). Alliance is considered a generic common factor, with meaning and usefulness expected to be preserved across theoretical orientations [2] and evidence suggests that a range of technology-based interventions can facilitate alliance [36]. Therefore, we hypothesized that the therapeutic relationship with a virtual therapist would correlate significantly with treatment outcome and offer insights on what components of the virtual therapist are most important for treatment efficacy.

## Methods

### Development and Description of the Virtual Therapist Alliance Scale

Inspiration for VTAS items came primarily from the WAI 12-item short report [37] and 32-item long report [1], covering dimensions of bond, task, and goal. Novel items were constructed using pre-existing items from across bond, task, and goal dimensions identified as most relevant to treatments conducted using a virtual therapist. All items were framed in reference to “the virtual therapist.”

Empathy is considered an important component of psychotherapeutic practice and has been shown to strengthen alliance through goals and tasks [38], yet it is not typically associated with artificial human actors. Therefore, categories for measuring empathic behavior from a study entitled *A Virtual Therapist That Responds Empathically to Your Answers* [34] were used to construct novel items.

Presence in virtual reality is the subjective experience of actually being in a virtual environment and that events are really occurring [16]; social presence, or copresence, is a specialized form of presence associated with the experience of *being with another* in a virtual environment [35]. Evidence suggests that presence may be a principle mechanism by which individuals experience emotions in a virtual environment, such as anxiety (eg, during exposure therapy [39]) or comfort and trust (eg, in the presence of a therapist [35]). The Social Presence Survey [40] was used to construct items relevant to work with a virtual therapist.

The resulting VTAS has a total of 17 items (see Table 1). Items were originally written in English, before translation into Swedish for data collection. Items were then retranslated into English and then back-translated into Swedish by experienced researchers and a psychotherapist to ensure accuracy. All disagreement was resolved by group consensus among bilingual researchers. All items are visually scored from 0 (*Do not agree at all*) to 4 (*Agree completely*) using the same response format and written anchors. The VTAS total scores were computed by summing all items.

### Samples and Procedure

Data for the VTAS psychometric evaluation were collected from two independent samples. The first study consisted of participants meeting criteria for spider phobia and randomized to a gamified VRET treatment, with virtual therapist support provided in Swedish [28]. Participants (n=50 eligible) received the intervention conducted within a single 3-hour period in the presence of a therapist (in case of severe emotional response), who was instructed to act as a computer technician. In the second study (Lindner et al, under review), participants (n=25 eligible) with similar severity of fear of spiders, most of whom also met criteria for spider phobia, were given a nearly identical VRET treatment, except the virtual therapist provided instruction in English. Instead of a technician being present with the patient, the patient received a set of steps to follow in order to complete VRET tasks and a number they could call for assistance.

**Figure 1:**
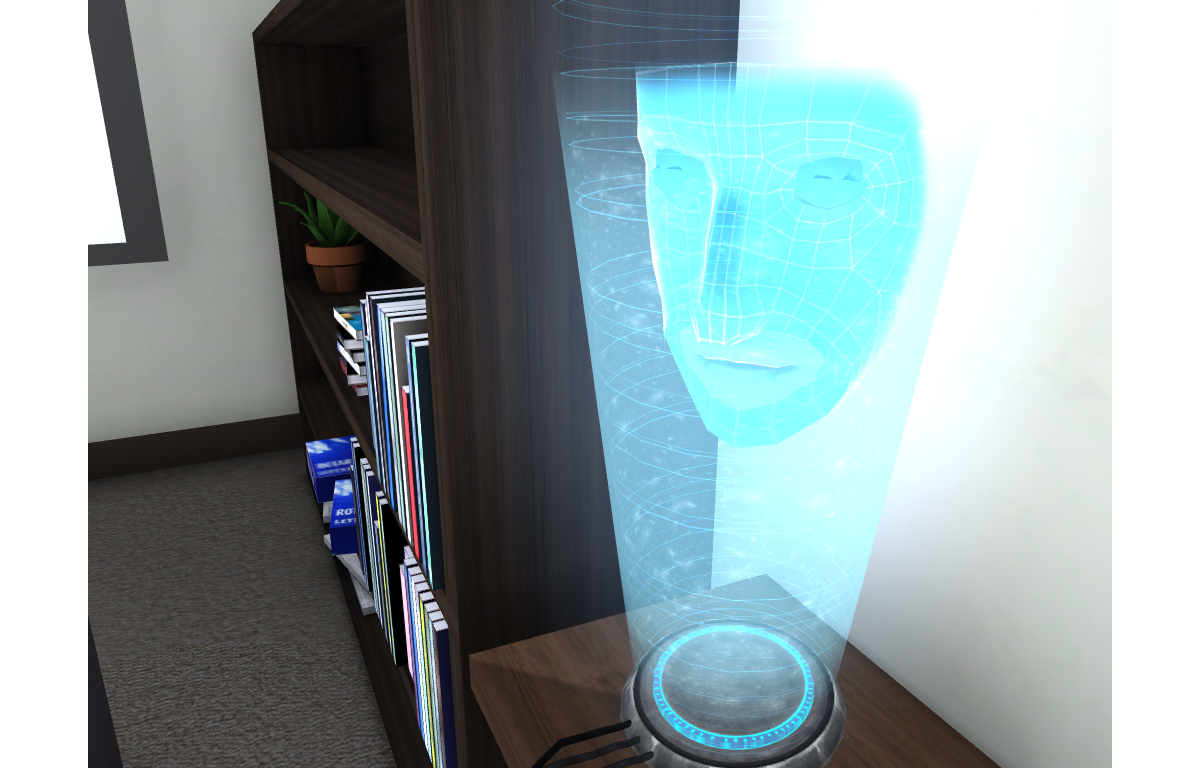
Physical embodiment of the primarily voice-based virtual therapist.

The virtual therapists in the two studies delivered psychoeducation, treatment instruction, reassurance during the course of a task, and reinforcement when a level was completed, primarily using voiceover instruction. In the second study, the virtual therapist was also given a physical embodiment by means of a holographic image that moved while speaking during the introductory phase of treatment (see Figure 1); instructions were complemented with animated graphics to emphasize psychoeducation lessons. Voiceover commentary was triggered at each stage of treatment in accordance with the initiation and completion of a task, as well as in accordance with participants’ Subjective Unit of Distress Scale (SUDS) ratings: if the SUDS rating by a participant went down over the course of a treatment stage, the patient was provided positive encouragement, and if the SUDS rating went up (ie, the patient became more fearful), the patient was informed that they could slow down and take more time if required. The voice of the virtual therapist was male in both studies and delivered instructions as “your therapist.” Both studies also included instances of an additional individual with a female voice referred to explicitly during treatment as a “spider expert,” who instead of providing therapeutic content, strictly delivered information about the biology and lifecycle of spiders. The language of instruction was Swedish and English in the first and second study, respectively.

All treatments were conducted in a university laboratory environment at Stockholm University. Participants were provided with inexpensive over-ear headphones and virtual reality devices—Gear VR (Samsung) using Galaxy S6 (Samsung) mobile phones—for use during the treatment. In the first study, the VTAS questionnaire was administered in paper format at the postassessment occasion (ie, 1 week following treatment). In the second study, the VTAS was administered along with other questionnaires immediately after treatment via an online survey. Outcome follow-ups were administered at 3 months in the first study and at 6 months in the second study. Outcome data were collected using an online tool [41] for all follow-up in the second study and for follow-up in the first study only if a participant was unavailable to meet in person.

VTAS surveys were not completed by 3 participants in the first study (2 dropouts prior to postassessment and 1 form never completed) and 2 participants in the second (both dropouts prior to treatment), giving a final sample of 70 participants with VTAS data. All items were mandatory, collected in the same order for all participants in both studies, and no individual items were missed. The Stockholm Regional Ethical Review Board approved both study 1 (2015/472-31) and study 2 (2018/1640-32). All participants provided written informed consent.

Regarding demographic characteristics and severity of symptoms, there was no difference in age between individuals in the first (mean 34.1, SD 10.9) and second study (mean 29.7, SD 11.0; t_73_=1.63, *P*=.11), nor differences in self-reported fear at baseline (t_73_=-1.32, *P*=.19). Participants were mostly female (42/50, 84%, and 19/25, 76%, respectively), with no significant difference between them (N=75, χ^2^_1_=0.7, *P*=.40).

### Other Measures

#### Fear of Spiders Questionnaire

The Fear of Spiders Questionnaire (FSQ) [42] is composed of 18 items and evaluates self-reported fear and avoidance of spiders. According to the authors, the FSQ has good internal consistency (Cronbach alpha=.92) and split-half reliability (.89).

#### System Usability Scale

The System Usability Scale (SUS) is a 10-item scale for assessing user friendliness [43] and appropriateness of a tool for a given context [44]; it is considered technology agnostic, showing utility across a range of interface types, including graphical and speech-based systems. The mean score on the SUS was 82.8 (SD 10.4) (n=70). The SUS was taken as a measure of convergent validity with bond and empathy; the SUS captured characteristics similar to alliance common factors, such as accessibility, empowerment, guidance, and delivery of a sense of security [45].

#### Igroup Presence Questionnaire

The Igroup Presence Questionnaire (IPQ) [46] is a scale used for measuring the subjective experience of being present in a virtual environment (ie, *presence*). The questionnaire is made up of 14 items, scored from -3 to +3; total scores are a summation of all items, with reverse-coded items compiled such that increased presence was always associated with positive numbers. Item 6 was removed from analysis due to a typo in printing. The mean score on the IPQ was 3.72 (SD 13.2) (n=47) and the Cronbach alpha was .874 (not including item 6). The IPQ was administered in the first study only. Presence according to the IPQ, and the Gatineau Presence Questionnaire (GPQ) below, were taken as a measure of convergent validity with VTAS copresence items.

#### Gatineau Presence Questionnaire

The GPQ [47] is a scale intended to measure the experience of presence in a virtual environment. In this study, the two positively worded items—“the impression of being there” (item 1) and “appraising the experience as being real” (item 2)—from the 4-item scale were used for analysis. The two negatively worded items (ie, being absent) were not included because they were not expected to correlate with other measures in this study. Items were rated from 0 to 10. The mean score on the first two items of the GPQ was 6.20 (SD 2.31) (n=23). These items had an adequate internal reliability (alpha=.77).

### Statistical Analyses

Data cleaning and processing was performed using SPSS for Windows, version 25.0 (IBM Corp), and analyses were conducted using the jamovi, version 1.0.5 (The jamovi project), statistical platform running on an R (The R Foundation) back end. Parallel analysis [48] was used to extract factor loading of items using maximum likelihood explorative factor analysis with oblimin rotation. Factors were identified based on factor loadings; in case of cross-loading (three items), the item was deemed associated with the factor it loaded most heavily on. As recommended by Clark and Watson [49], a sensitivity analysis was carried out that removed similarly cross-loading items, with the threshold set at 0.20 (two items above threshold). VTAS factors used sum scores when conducting correlations with other measures but mean scores when conducting *t* tests. Cronbach alpha and McDonald omega were calculated as measures of internal consistency. Convergent validity was assessed by comparing the VTAS to presence and user-friendliness scores using Pearson correlations. Presence scores were z-transformed since the two studies used different measures. The Steiger test was used to evaluate differences in correlations with other measures between VTAS factors 1 and 2 [50]. Change scores on the symptomatology outcome measure were calculated by subtracting the later time period from the earlier time period (eg, post minus pre). Next, bivariate correlations were conducted between VTAS scores and symptoms change (pre-post and post-follow-up, respectively), presence, and user-friendliness scores. Finally, separate multiple regression models included all these predictors at once, examining the unique associations to VTAS total and subscale scores, while holding the other variables constant. Missing data were handled with case-wise deletion.

## Results

### Effect of Demographics

**Figure 2:**
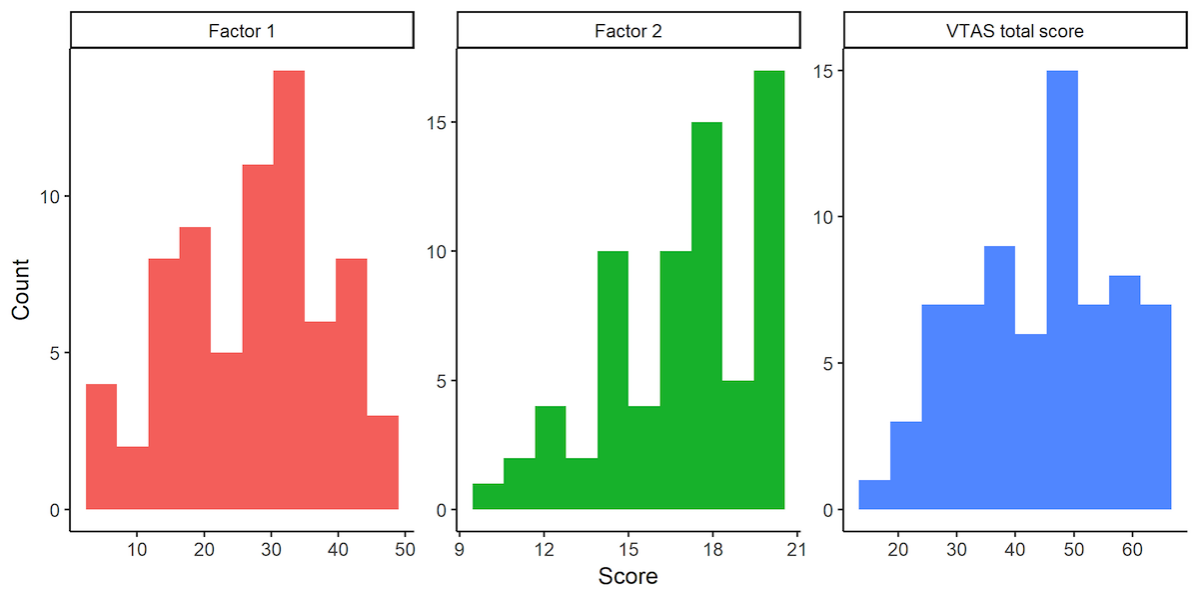
Histograms of the Virtual Therapist Alliance Scale (VTAS) total and subscale scores.

There was no effect of sex on VTAS scores (F_1,68_=.269, *P*=.61), nor correlations with age (*r*=-.095, *P*=.43). Mean VTAS scores were 44.0 (SD 12.2) in the first study and 45.0 (SD 13.4) in the second study; VTAS scores followed an approximate normal distribution as evaluated by visual inspection of the VTAS total score histogram (see Figure 2) and computation of the Shapiro-Wilk test (*P*=.11).

### Exploratory Factor Analysis

**Figure 3:**
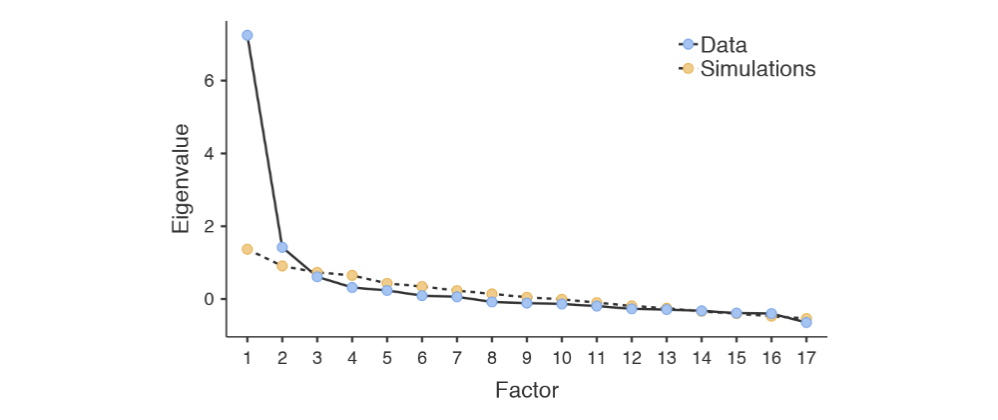
Parallel analysis scree plot of Virtual Therapist Alliance Scale (VTAS) items.

Evaluation of a parallel analysis scree plot for the VTAS indicated a clear, marked reduction of eigenvalues after the first factor, followed by another small reduction after the second factor, remaining consistent thereafter (see Figure 3). This suggests a two-factor solution for the VTAS. Factor 1 consisted of 12 items from task, goal, and copresence categories (except item 3), and factor 2 consisted of five items from bond and empathy (except item 11). Factor 1 explained 38.8% of total variance in the sample and factor 2 explained 14.0% of total variance. All factors had loadings above 0.35—the cutoff recommended by Clark and Watson [49]—and just one item (from factor 2) loaded below 0.40 (item 4, 0.363; see Table 1). The Pearson correlation between factor 1 and factor 2 sum scores was moderate and significant (*r*=.493, *P*<.001), and there was a significant difference between factors 1 and 2 mean scores, with the latter scoring higher (mean -1.13, SE 0.096; t_69_=-11.8, *P*<.001).

**Table 1:** Factor loadings for the two factors, including mean item-level scores.

Item	Factor loadings^a^		Mean (SD)
	Factor 1	Factor 2	
1. I experienced the virtual therapist as friendly		0.778	3.81 (0.46)
2. I experienced the virtual therapist as warm		0.643	3.27 (0.90)
3. I felt that the virtual therapist gave clear instructions		0.614	3.63 (0.62)
4. I experienced the virtual therapist as supportive		0.363	3.24 (0.94)
5. The presence of the virtual therapist made the experience more enjoyable	0.492	0.437	3.14 (0.92)
6. It felt like the virtual therapist shared the virtual environment with me	0.580		2.50 (1.28)
7. The virtual therapist appeared alive to me	0.549	0.307	2.19 (1.18)
8. I felt that the virtual therapist and I interacted	0.695		1.41 (1.12)
9. The way that the virtual therapist communicated was captivating	0.659		1.76 (1.21)
10. I felt that the virtual therapist was trustworthy	0.427	0.465	3.04 (0.96)
11. It felt comforting to have the virtual therapist there with me	0.791		2.33 (1.37)
12. The presence of the virtual therapist helped me achieve my goals	0.910		2.30 (1.34)
13. The virtual therapist and I shared common goals	0.648		2.47 (1.21)
14. I felt that the virtual therapist understood my fears	0.710		2.73 (1.17)
15. I felt that the virtual therapist tailored the treatment according to my needs and progress	0.808		1.60 (1.15)
16. The encouragement of the virtual therapist helped me	0.969		2.26 (1.26)
17. The virtual therapist gave me new perspectives on my troubles	0.601		2.61 (1.28)

^a^The *maximum likelihood* extraction method was used in combination with an *oblimin* rotation.

A sensitivity analysis was conducted by removing cross-loaded items. In this case, factors 1 and 2 correlated less strongly (*r*=.320, *P*=.007) and the factor 2 mean score continued to be higher than that of factor 1 (mean -1.29, SE 0.11; t_69_=-11.7, *P*<.001).

### Internal Consistency

The VTAS total Cronbach alpha was .921 (with identical McDonald omega) and was similar across the two subsamples (study 1 alpha=.915, omega=.917; study 2 alpha=.936, omega=.938). Factor 1 Cronbach alpha and McDonald omega was .931 and .932, respectively. This was higher than factor 2, which had Cronbach alpha=.710 and McDonald omega=.759. Factor 2 internal consistency was not improved by dropping the lowest loading item (item 4: alpha=.670, omega=.738). A sensitivity analysis conducted with cross-loaded items removed indicated an internal consistency of alpha=.928 and omega=.930 for factor 1 and alpha=.694 and omega=.751 for factor 2.

### Convergent Validity

Presence scores (z-standardized), including data from both studies and assessing convergent validity, correlated strongly with VTAS total scores (*r*>.50; see Cohen 1988 as cited in Hemphill [51]; see Table 2). Factor 1, which included all copresence items, also correlated strongly with presence, while factor 2, which contained no copresence items, had only a weak correlation. A Steiger test indicated a significant difference between the two factors in regard to correlations with presence.

User-friendliness scores, as measured by the SUS, were found to correlate moderately with VTAS total scores. Factor 1 correlated weakly with user friendliness, whereas factor 2 correlated moderately. There was no significant difference, however, between factor correlations with user friendliness according to the Steiger test. A sensitivity analysis conducted with cross-loaded items removed indicated a similar pattern of results with no changes in significance levels.

**Table 2:** Correlations table between Virtual Therapist Alliance Scale (VTAS) total and factor sum scores as compared to process measures, outcome difference scores, and Steiger test conducted between factor correlations.

Measure	VTAS total			Factor 1 sum			Factor 2 sum		Steiger test		
	*r*	*P*	*r*		*P*	r		*P*		z	*P*
SUS^a^	.351	.003	.300		.01	.407		<.001		-0.844	.40
Presence *z*-score	.592	<.001	.603		<.001	.298		.01		3.292	<.001
FSQ^b^ pre-post	-.213	.08	-.216		.08	-.107		.39		0.720	.47
FSQ post-follow-up	-.310	.01	-.333		.007	-.078		.54		1.824	.07

^a^SUS: System Usability Scale.

^b^FSQ: Fear of Spiders Questionnaire.

### Correlations Between Fear of Spiders Questionnaire Change Scores and Virtual Therapist Alliance Scale

Correlations were conducted to evaluate the relationship between VTAS total scores and change in FSQ at postassessment and over follow-up (see Table 2). At postassessment, the VTAS did not correlate significantly with symptom change scores; however, over the follow-up period, the VTAS correlated moderately with change scores.

Neither VTAS factor 1 nor factor 2 sum scores correlated significantly with FSQ change scores pre- to posttreatment. Over follow-up, however, factor 1 moderately correlated with change scores, whereas factor 2 did not and was not significant. Steiger tests did not determine a significant difference of correlations between factor 1 and factor 2 during either change-score time period (*P*>.07 for both). A sensitivity analysis conducted without cross-loaded items identified a similar pattern of results with factor 1 correlating significantly with outcome only over follow-up (*r*=-.338, *P*=.006 versus *r*=-.217, *P*=.08), and factor 2 not correlating with outcome at either time outcome (*P*>.45 for both).

### Prediction of Virtual Therapist Alliance Scale Scores

A multiple regression analysis was used that included all predictors of VTAS total scores in a single model (see Table 3). Due to case-wise deletion and missing data across three time periods, a total of 64 out of 70 (91%) participants were included. Of the four covariates, user friendliness, presence, and follow-up FSQ change scores were all significantly associated with the VTAS. Two additional models were included, with each factor as a dependent variable. In the model with factor 1, only presence and follow-up FSQ change scores significantly predicted VTAS factor 1 sum scores; in the model with factor 2, only user friendliness was significant.

**Table 3:** Multiple linear regression table of Virtual Therapist Alliance Scale (VTAS) total and factor sum values as dependent variables, with included process measure and outcome difference score covariates.

Measure	VTAS total			Factor 1 sum			Factor 2 sum			
	B (SE)	*P*	B (SE)		*P*	B (SE)		*P*		
Intercept	14.848 (10.584)	.17	7.248 (9.312)		.44	7.600 (2.630)		.005		
SUS^a^	0.311 (0.124)	.02	0.203 (0.109)		.07	0.108 (0.031)		<.001		
Presence z-score	5.949 (1.454)	<.001	5.590 (1.280)		<.001	0.359 (0.361)		.32		
FSQ^b^ pre-post	-0.097 (0.065)	.14	-0.084 (0.057)		.15	-0.014 (0.016)		.41		
FSQ post-follow-up	-0.183 (0.069)	.01	-0.177 (0.061)		.005	-0.006 (0.017)		.74		

^a^SUS: System Usability Scale.

^b^FSQ: Fear of Spiders Questionnaire.

## Discussion

Automated VRET treatments using embodied virtual therapists may be a means of increasing access to exposure therapy by supporting patients to conduct treatments on their own; however, little has been known about patient alliance toward the virtual therapist and its relationship to treatment outcome. This study evaluated a novel scale—the VTAS—using data from two samples of spider-fearful patients treated over a single session.

Psychometric evaluation of the VTAS identified a two-factor solution for the scale, with items based on task, goal, and copresence loading primarily on factor 1, and items based on bond and empathy on factor 2. As summarized in Hatcher and Gillaspy [37], a two-factor solution for the WAI scale is not uncommon. Falkenström, Hatcher, and Holmqvist [52] conducted a confirmatory factor analysis of the scale among three large samples in Sweden and the United States and argued that, given the high intercorrelation between task and goal factors, a two-factor solution was more defensible psychometrically. A recently evaluated WAI scale for guided Internet-based treatments had a similar task-goal and bond two-factor solution [53]. Goal and task factors have been referred to as *agreement-confidence* in the therapist and affective bond as *relationship* [54]. While this terminology may not be appropriate for use with a virtual therapist, if such a therapist can be conceived of in terms of a guide motivating the patient toward behavior change, then a suitable terminology could be task-oriented versus relationship-oriented guidance [55].

It should be noted, however, that not all goal and task items loaded on the first factor and vice versa. For example, item 11—“It felt comforting to have the virtual therapist there with me,” which suggests a bond category—loaded quite highly on factor 1 (0.791), and item 3—“I felt that the virtual therapist gave clear instructions,” suggesting a task category—loaded quite highly on factor 2 (0.614). In the context of using a foreign technical application (ie, VRET), a relationship between clarity of instruction and development of affective bond could be suggested by this outcome (item 3 in factor 2); also, a relationship between receiving comfort and aid and accomplishing the tasks and goals of therapy (item 11 in factor 1). Alternatively, identification of the factor structure could be based on how items were written. Factor 2 items, for the most part, attributed a human quality to the virtual therapist—“I felt/experienced that the virtual therapist was...” *friendly* (item 1), *warm* (item 2), *supportive* (item 4), *trustworthy* (item 10), and *gave clear instructions* (item 3)— whereas factor 1 items were all written as an observation on the nature of the relationship or the benefits accruing from the presence of the virtual therapist. Previous authors of text-based, self-help treatments have argued that alliance is toward the application, as summarized in Heim et al [30]; however, embodied avatars do contain more realistic, human-like qualities and, therefore, the concept of alliance here may be closer to the traditional one. Further research will be needed, such as comparing different forms of embodiment (eg, audio-only instruction versus audio plus physical embodiment), using a rating scale sensitive to this. Preliminary results from this study suggest patients rate factor 2 bond items highly (ie, significantly higher than those of factor 1, *P*<.001); also, factor 2 items explain a sizeable additional total variance in the scale over five items (38.8% vs 14.0%). It is conceivable that bond is strengthened in interventions with an embodied avatar as compared to text-based, self-help applications [35] or, as suggested by Heim et al [30], bond may occur earlier in treatment. In certain treatments, low bond (ie, trust and faith) may be sufficient, whereas in other treatments, such as exposure therapy, high bond may be needed to motivate treatment completion.

Relational bond between patient and mobile self-help apps was explored recently in a mixed qualitative-quantitative study [33]. Qualitative analysis suggested patients do form a personal bond even with their nonavatar, text-based app, referring to it as a “therapist in their pocket” and a friend in the app they could turn to for reassurance and encouragement. Quantitative analysis using a purpose-built questionnaire for measuring alliance in self-guided programs—the mobile Agnew Relationship Measure (mARM)—evaluated one iteration of their scale that simply exchanged “therapist” for “app” on all items of the Agnew Relationship Measure (ARM). Their findings suggest that while certain item terminology could risk inappropriately anthropomorphizing an app—therefore, making it harder to relate to—where human-like qualities are concerned, participants are more likely to endorse a relationship item that includes a qualifying perception (eg, “I feel the app...”) rather than a statement of fact (eg, “The app seems bored or impatient with me”). The former was done for all items attributing human characteristics to the virtual therapist in this study. Internal consistency was lower on factor 2 (Cronbach alpha=.710) and below recent cutoff recommendations by Clark and Watson (alpha=.80) [49]; however, a high coefficient alpha is difficult to obtain in scales with few items, given its relationship to length (ie, 12 versus five items on factor 2) [49]. Factor 1 and overall VTAS consistency was high (Cronbach alpha=.931 and .921, respectively).

Convergent validity findings provide additional support for the independence of factors. The demographic variables age and gender did not have a significant relationship with VTAS scores (*P*>.43 for both). Questionnaires capturing the subjective experience of presence in a virtual environment were found to correlate significantly more with factor 1 (*P*<.001), in which all copresence items loaded. Scores of user friendliness correlated moderately with both factor 1 and factor 2 (*P*<.01 for both). However, of the two measures, only presence significantly predicted factor 1 VTAS results in a multiple linear regression model, other than FSQ follow-up scores, and only user friendliness predicted factor 2 VTAS results. It should be noted that user-friendless scores were between good (71.4) and excellent (85.5), according to generic SUS adjective descriptors (mean 82.8) [56]. This is positive, considering that good system usability may be particularly important in automated treatments that are intended to be self-administered [29].

Evidence for the relationship with outcome indicates that perceived alliance toward a virtual therapist had a small, nonsignificant (*r*=.213) correlation with outcome at posttreatment, but a moderate and significant (*r*=.310) correlation at follow-up. Unlike the study by Heim et al [30], which showed a correlation between bond-factor and outcome and is the only other known study to measure alliance to a therapist avatar in an automated treatment format, only factor 1 (ie, task, goal, and copresence) correlated with outcome in this study. The lack of significant relationship with outcome immediately after treatment is not surprising, given the small-to-moderate sample size and limited power to detect a small association: a post hoc power analysis indicated that 70 participants would have 80% power to detect a correlation of above .33. However, the significant relationship at follow-up may suggest that those who had a good alliance with the virtual therapist better understood and interpreted the treatment instructions correctly or received adequate reinforcement to apply them in their daily lives. Further research is needed here, as well as a better understanding of why particular factors correlate with outcome (eg, as compared to Heim et al [30]). A recent narrative review of therapeutic alliance in Internet interventions found no other study that identified a relationship between the bond-factor and outcome, but many with a meaningful and statistically significant relationship between task-goal and outcome [9]. The author suggests that this may be due to a ceiling effect from high scores on the bond dimension in Internet interventions, similar to this study, or possibly the relative lack of importance of bond in treatments with little-to-no face-to-face interaction. Nevertheless, overall results in this study compare favorably to current meta-analytic best evidence for the relationship between alliance and outcome [57]. Across 39 different alliance measures and 306 studies, the alliance correlation with outcome was .278 (*P*<.0001); over 18 Internet-based studies, the alliance correlation with outcome was .275 (*P*<.0001).

Limitations of this research include the relatively small sample size in the two studies included and small differences between the automated treatments, such as language used and whether the treatments were carried out with a technician present. Together, this meant that measurement invariance had to be assumed but could not be formally tested. The use of a single-session treatment format prevented the exploration of cross-lagged and mediation models to predict symptom reduction following a session [58]. This exploration is important, given the limited capacity of alliance research to show an experimentally causal association with treatment outcome [57]. In the Heim et al [30] study, multiple treatment sessions also provided the authors evidence that a human therapist was increasingly missed over time, although this may be due to the therapeutic techniques used by the virtual avatar in later sessions. Finally, this study explored only client-reported ratings of alliance, versus therapist, observer, and friend or family ratings, whereas other studies, such as a recent blended Internet-based CBT treatment for depression, found only therapist-rated alliance results to be significant with outcome [59].

In the future, research on automated treatments may benefit from evaluating different forms of embodiment for virtual therapists and exploring their effect on alliance, as mentioned earlier, and relationship to treatment outcome and treatment adherence. The virtual therapist in this study primarily used voice-based instruction and was noninteractive; however, it is conceivable that a quality implementation of visual presence, behavioral realism, and interactivity may improve outcomes [35]. Heim et al [30] suggest this may be possible through improved credibility and expectancy, which mediation models indicate function in a bidirectional relationship with alliance to improve outcomes [60]. Previous research on embodied graphically generated agents have included SimCoach [61], which used rich visual representations of humans, gestures, and emotions, as well as natural conversational speech to comfort, guide, and motivate armed service members toward psychological health and to solicit anonymous information. As summarized by Lucas et al [62], rapport may be improved by relying on verbal and nonverbal behavior, such as specific verbal patterns, intonation, and choice of language as well as welcoming gestures, open posture, and attentive eye gaze. Disclosure of personal backstories and intimate personal details by an automated therapist has been shown to increase self-disclosure by highly anxious patients [63]. Relational behaviors, such as calling the patient by name, use of humor when appropriate, including information discussed during past interactions, and engaging in a social chat at the beginning of a new interaction, have all been shown to increase how much a patient feels cared for and how useful the information they received felt to them [25]. Adherence to psychoeducation, operationalized as successful task execution, may be improved by attitude toward a virtual agent, such as their level of trust of the agent, its realism, and its amiableness [64].

In conclusion, this is the first study evaluating an alliance scale for use with virtual therapists in an automated VRET treatment format. Preliminary evidence suggests that this novel VTAS instrument has sound psychometric properties, with one exception being the somewhat low internal consistency of factor 2. Nevertheless, despite this and other limitations, alliance toward a virtual therapist was found to be positively correlated with treatment outcome at follow-up. Further exploration of the predictive abilities of this scale on treatment outcome and adherence is warranted, particularly in conjunction with alternative forms of embodiment, in larger samples of patients, and across different treatment programs and treatment formats.

## Abbreviations

ARET: augmented reality exposure therapy
ARM: Agnew Relationship Measure
CBT: cognitive behavioral therapy
FSQ: Fear of Spiders Questionnaire
GPQ: Gatineau Presence Questionnaire
HAq: Helping Alliance Questionnaire
ICBT: Internet-based cognitive behavioral therapy
IPQ: Igroup Presence Questionnaire
mARM: mobile Agnew Relationship Measure
SUDS: Subjective Units of Distress Scale
SUS: System Usability Scale
VR: virtual reality
VRET: virtual reality exposure therapy
VTAS: Virtual Therapist Alliance Scale
WAI: Working Alliance Inventory
WAI-S: Working Alliance Inventory-short form
WAI-VAR: Working Alliance Inventory applied to virtual and augmented reality
